# Unveiling the heterogeneity: PD-L1 expression and metabolic activity in primary vs. metastatic NSCLC, a retrospective single-center analysis

**DOI:** 10.3389/fonc.2026.1966803

**Published:** 2026-10-13

**Authors:** Bingxin Hu, Huibin Jin, Yue Yu, Jie Zhang, Yang You, Chang Fu, Ang Xuan, Xinyu Wu, Yongju Gao

**Affiliations:** 1 Department of Nuclear Medicine of Henan Provincial People’s Hospital & the People’s Hospital of Zhengzhou University, Zhengzhou, China; 2 Henan Key Laboratory of Novel Molecular Probes and Clinical Translation in Nuclear Medicine, Zhengzhou, China

**Keywords:** PD-L1, NSCLC, primary tumors, metastatic tumors, SUVmax

## Abstract

**Introduction:**

Programmed death-ligand 1 (PD-L1) expression was different in primary tumors (PT) and metastatic tumors (MT). Total-body positron-emission tomography/computed tomography (PET/CT) comprehensively visualizes primary lesions and metastases. We aimed to explore the heterogeneity of PD-L1 high expression and metabolic activity between primary and metastatic lesions in patients with non-small cell lung cancer (NSCLC).

**Methods:**

Immunohistochemistry analysis (IHC) was conducted for PD-L1 expression on tumor cells (TC) and tumor-infiltrating immune cells (IC) with 22C3 antibody. PD-L1 positive scores on TC no less than 50% or IC no less than 10% was defined as high expression. Total 598 (456 adenocarcinoma (ADC) and 142 squamous cell lung carcinoma (SCC)) primary diagnosed NSCLC patients who underwent ^18^F-FDG-PET/CT imaging were collected retrospectively. The statistic methods included chi-square test, Mann Whitney test and multivariate regression.

**Results:**

598 patients included 441 with PD-L1 low expression and 157 high expression. In all subtypes and ADC, MT had higher PD-L1 high expression (P = 0.023 and P = 0.010, respectively). The maximum standard uptake value (SUVmax) of PD-L1 high expression was significantly higher than that of PD-L1 low expression, no matter in PT or MT (15.23 (8.85) vs 9.42 (8.34), P<0.001; 13.36 (5.55) vs 8.52 (6.93), P<0.001). The squamous-cell-carcinoma metastatic-lesion analyses were exploratory owing to the very small sample size and risk of type-II statistical error. Multivariate regression indicated that SUVmax was independently associated with PD-L1 high expression. In all metastatic sites, we compared the SUVmax of MT and paired PT and found SUVmax was higher in PT than MT in PD-L1 low expression subgroup (11.39 (8.12) vs 8.52 (6.93), P = 0.002). For lymph node metastases, SUVmax was significantly elevated in PT compared to MT in PD-L1 low expression (13.71 (7.95) vs 8.54 (7.30), P = 0.002), whereas no correlation was observed in PD-L1 high expression.

**Discussion:**

Both PD-L1 expression and metabolism exhibited heterogeneity between primary tumors and their metastases. Compared to the primary tumors, different types of metastatic tumors showed variations not only in PD-L1 expression but also in their metabolic parameter. Utilizing whole-body PET/CT imaging facilitates the selection of optimal biopsy sites by providing metabolic reference data pertinent to clinical practice.

## Introduction

Clinical trials have demonstrated that immunotherapy significantly improves the prognosis of patients with advanced (stage III-IV) non-small cell lung cancer (NSCLC) (1–5). According to guidelines from the EMA and FDA (6, 7), patients with advanced-stage lung cancer and high programmed death-ligand 1 (PD-L1) expression may receive immunotherapy as first-line treatment. PD-L1 high expression was defined as positive expression either on tumor cells no less than 50% or immune cell expression no less than 10%. Currently, immunohistochemistry is the most commonly used method for PD-L1 detection (6, 7). Some previous studies (8, 9) concluded PD-L1 had higher expression in PT than that in MT, while the others (10, 11) had opposite conclusions.

As a standard diagnostic and staging tool in oncology, Total-body positron-emission tomography/computed tomography (PET/CT) comprehensively visualizes primary lesions and metastases (12, 13). Compared to conventional imaging and random biopsy, total-body PET/CT integrates anatomical and metabolic information, enabling precise localization of metabolically active tumor lesions, minimizing sampling errors, and providing a more reliable lesion basis for paired analysis of primary and metastatic tumors. Studies have shown that the metabolic parameters (including standard uptake value (SUVmax), the ratio of PT-SUVmax and SUVmax of liver or blood pool (SUR-L, SUR-BP)) are correlated with PD-L1 expression in both NSCLC, adenocarcinoma (ADC) and squamous-cell-carcinom (SCC) (14, 15).

Previous studies rarely analyzed metabolic differences between PT and MT. Given the variability of PD-L1 expression in PT and MT, coupled with its association with tumor metabolism, we conducted a retrospective analysis to investigate the heterogeneity of PD-L1 expression and SUVmax in both primary and metastatic lesions of lung cancer patients.

## Material and methods

### Patients

Totally 598 patients were enrolled with primary NSCLC from January 2021 to January 2024 at our hospital. Inclusion criteria included the first diagnosis of NSCLC without previous systemic disease or treatment, complete pathological data, and available whole-body PET/CT images before PT or MT biopsy (within two weeks). The clinical trial registration is not applicable for retrospective observational study.

### ^18^F-FDG PET/CT

All patients were required to fast for at least 6 hours and their serum glucose levels were not higher than 10 mmol/L before intravenous injection of ^18^F-FDG with a dosing regimen (3.7 MBq/kg). Following an approximate 50-minute rest period after injection, each patient underwent PET/CT imaging. All images were acquired on the whole-body PET/CT (uEXPLORER, Shanghai United Imaging Medical Technology, China). First, a low-dose CT scan (tube current 10 mA, voltage 100 kV, rotation time 0.5 s, pitch 1.0125, collimation 80 × 0.5 mm) was performed for anatomical positioning and reconstructed in a 512 × 512 matrix for attenuation correlation analysis. The reconstruction algorithm was List-Mode Time-of-Flight Ordered Subsets Expectation Maximization (List-Mode TOF-OSEM). Second, 5-minute PET imaging (4 iterations and 20 subsets) was conducted. Third, Two experienced nuclear medicine physicians analyzed the images blinded to the pathological results and clinical information of patients, manually marked the regions of interest (ROI) on the primary lesions and the metastatic lesions, and collected the maximum standard uptake value (SUVmax). For patients with metastatic biopsy sites, the SUVmax value of their paired primary tumors were simultaneously collected. All SUVmax values were strictly retrospectively measured based on biopsy-proven malignant lesion. If the two physicians’ assessments differ, a third person served as tie-breaker for final adjudication.

### Immunohistochemical staining

All tissues from PT or MT were stained on an automated stainer with 22C3 (PD-L1 test kits, DAKO/Agilent, USA) (16). PD-L1 expression on TC was evaluated and scored with number of PD-L1 positive tumor cells/(Total number of PD-L1 positive + PD-L1 negative tumor cells) × 100%. It was the same to the expression on IC. PD-L1 positive scores on TC no less than 50% or IC no less than 10% was defined as high expression, satisfying either criterion; only lesions meeting neither criterion were classified as low-expression, based on previous clinical trials (1, 2). This represents a local exploratory composite definition applied in our single-center retrospective cohort, not a validated companion-diagnostic criterion.

### Statistic

In all tests, two sided P<0.05 were defined as statistical significance. In order to screen candidate variables associated with PD-L1 expression, the chi-square test was used for categorical variables, Mann-Whitney test for continuous variables and Wilcoxon signed-rank test for paired within-subject comparisons of matched primary-metastatic lesions. Multivariate regression was performed to explore the independent factors associated with PD-L1 expression. Odds ratios and 95% confidence intervals (95%CI) were calculated. The odds ratio (OR) represents the odds of PD-L1 high-expression relative to PD-L1 low-expression for each one-unit increment in SUVmax. To mitigate the risk of type I error inflation caused by multiple testing across univariate associations (**Table 1**) and multivariate logistic regression predictors (**Table 2**), we implemented the Benjamini-Hochberg false-discovery-rate (FDR) correction. Both raw p-values and FDR-adjusted q-values were presented in the corresponding tables. Statistical significance after multiple-comparison correction was defined as q-value < 0.05. The SUVmax values were not normally distributed, so the median (interquartile range, IQR) was used for representation. All statistically analyses were conducted using SPSS V23.0 and R software V4.3.1.

**Table 1:** Clinicopathological characteristics.

	All subtypes					ADC					SCC				
Characteristics	Low	High	χ^2^	P	q	Low	High	χ^2^	P	q	Low	High	χ^2^	P	q
Age (year)			0.000	0.983	0.983			1.880	0.170	0.204			2.108	0.147	0.774
<63	199	71				166	59				33	12			
≥63	242	86				183	48				59	38			
Gender			22.436	**<0**.**001**	**<0.001**			16.516	**<0.001**	**<0.001**			0.599	0.439	0.774
Male	233	117				150	70				83	47			
Female	208	40				199	37				9	3			
Diameter (mm)			10.560	**0.001**	**0.002**			6.659	**0.010**	**0.015**			1.157	0.282	0.774
<30	218	54				190	43				28	11			
≥30	223	103				159	64				64	39			
Smoking history			20.898	**<0**.**001**	**<0.001**			21.450	**<0.001**	**<0.001**			0.213	0.645	0.774
Non-smoker	278	66				259	54				19	12			
Smoker	163	91				90	53				73	38			
Bx site			5.191	**0.023**	**0.034**			6.602	**0.010**	**0.015**			0.301	0.583	0.774
Primary	375	121				293	78				82	43			
Metastatic	66	36				56	29				10	7			
Metastatic sites			2.175	0.704	0.844			2.276	0.685	0.685			0.073	0.787	0.787
Lymph node	44	25				35	19				9	6			
Bone	13	6				12	5				1	1			
Brain	2	3				2	3								
Pleural fluid	4	1				4	1								
Pleura	3	1				3	1								

Bold, P<0.05 or q<0.05; q, values calculated using the Benjamini-Hochberg false-discovery-rate correction for multiple comparisons; Low, PD-L1 low expression; High, PD-L1 high expression; ADC, adenocarcinoma; SCC, squamous cell carcinoma.

**Table 2:** Multivariate regression on PD-L1 expression.

	All subtypes			ADC			SCC		
Characteristics	OR (95%CI)	P	q	OR (95%CI)	P	q	OR (95%CI)	P	q
Gender		0.062	0.124		0.183	0.229			
Male									
Female	0.581 (0.329-1.027)			0.653 (0.348-1.223)					
Diameter (mm)		0.877	0.877		0.826	0.826			
<30									
≥30	1.035 (0.673-1.590)			0.945 (0.569-1.569)					
Smoking history		0.337	0.404		0.054	0.090			
Smoker									
Non-smoker	1.300 (0.761-2.222)			1.839 (0.990-3.417)					
Bx site		0.028	0.084		0.030	0.075			
Primary									
Metastatic	1.740 (1.061-2.855)			1.843 (1.060-3.202)					
Pathological subtypes		0.202	0.303						
ADC									
SCC	0.719 (0.433-1.194)								
Metabolic activity		<0.001	<0.001		<0.001	<0.001		0.056	0.056
SUVmax	1.128 (1.089-1.168)			1.118 (1.074-1.164)			1.037 (0.999-1.077)		

Bold, P<0.05 or q<0.05; q, values represent Benjamini-Hochberg false-discovery-rate adjusted values for multiple-testing correction; OR, Odds ratios, the odds of PD-L1 high-expression relative to PD-L1 low-expression for each one-unit increment in SUVmax; ADC, adenocarcinoma; SCC, squamous cell carcinoma.

*, P<0.05; CI, confidence intervals; ADC, adenocarcinoma; SCC, squamous cell carcinoma.

## Results

### Patients’ clinicopathological characteristics

A total of 598 patients from 441 expressing PD-L1 low and 157 high were analyzed, shown on **Table 1**. In our research, male (33.43% vs 16.13%, P<0.001), diameter no less than 30mm (31.60% vs 19.85%, P = 0.001) and smoker (35.83% vs 19.19%, P<0.001) had higher PD-L1 expression. Metastatic sites were more likely to be PD-L1 high expression compared with primary sites (35.29% vs 24.40%, P = 0.023). The metastatic sites included lymph nodes, bones, brain, pleural fluid and pleura, and there was no significant difference in PD-L1 expression among them.

In adenocarcinoma (ADC) patients, there were 349 patients with low PD-L1 expression and 107 patients with high expression. Following factors had higher probability of PD-L1 high expression: male (31.82% vs 15.68%, P<0.001), diameter no less than 30mm (28.70% vs 18.45%, P = 0.010), smoker (37.06% vs 17.25%, P<0.001), and metastatic sites (34.12% vs 21.02%, P = 0.010).

In squamous cell carcinoma (SCC) patients, there were no positive factors.

After Benjamini-Hochberg FDR correction, those related factors remained significantly differences, shown **Table 1**.

### SUVmax and PD-L1 expression in primary and metastatic tumors

PD-L1 results derived from primary or metastatic sites were matched with PET parameter obtained from the corresponding primary or metastatic sites, respectively, shown on **Figure 1**.

**Figure 1:**
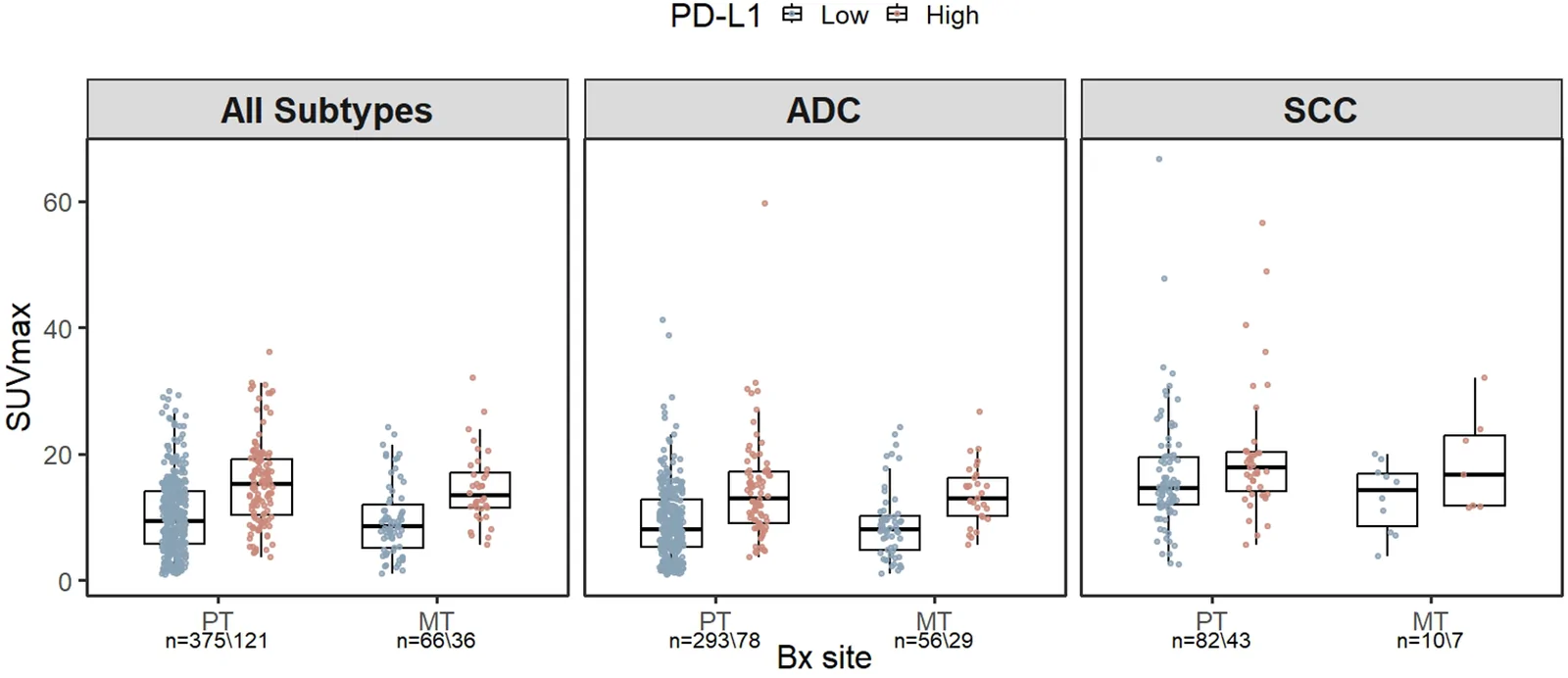
SUVmax and PD-L1 expression(the two obtained from the identical anatomical lesion) in primary and metastatic tumors: In all subtypes, SUVmax of PD-L1 high expression was significantly higher than that of PD-L1 low expression, in both PT and MT; In ADC, SUVmax of PD-L1 high expression was significantly higher than that of PD-L1 low expression, in both PT and MT; In SCC, SUVmax was significantly elevated in PD-L1 high expression compared to PD-L1 low cases in PT, whereas no correlation was observed in MT.

In all subtypes, the SUVmax of PD-L1 high expression was significantly higher than that of PD-L1 low expression, in both PT (15.23 (8.85) vs 9.42 (8.34), P<0.001) and MT (13.36 (5.55) vs 8.52 (6.93), P<0.001).

In ADC, SUVmax was higher in PD-L1 high than low expression, including PT (13.00 (8.13) vs 8.06 (7.52), P<0.001) and MT (13.02 (6.10) vs 8.10 (5.35), P<0.001).

In SCC, SUVmax was significantly elevated in PD-L1 high expression compared to PD-L1 low cases in PT (17.78 (6.09) vs 14.65 (7.63), P = 0.019), whereas no correlation was observed between SUVmax and PD-L1 expression in MT (16.74 (11.21) vs 14.23 (8.37), P = 0.193).

In all subgroups, there was no significant difference in SUVmax between PT and MT.

### SUVmax and PD-L1 expression in paired primary and metastatic tumors

All individual factors influencing PD-L1 high expression were incorporated into the multivariate regression analysis. In multivariate logistic regression, SUVmax was the sole related factor of high PD-L1 expression after Benjamini-Hochberg FDR correction, in both NSCLC (OR 1.128, 95%CI 1.089-1.168, raw P<0.001, q<0.001) and ADC (OR 1.118, 95%CI 1.074-1.164, raw P<0.001, q<0.001). Meanwhile, biopsy lesion origin was related to PD-L1 expression in NSCLC and ADC groups, shown on **Table 2**.To assess the association between PD-L1 expression and metabolic heterogeneity, SUVmax values of MT and matched PT were compared in patients with pathologically confirmed metastatic sites, shown on **Table 3**.

**Table 3:** The relationship between PET parameter and PD-L1 expression in paired PT-MT.

PD-L1 expression	SUVmax (median (IQR))							
	Low				High			
	PT	MT	Z	P	PT	MT	Z	P
All metastatic sites	11.39 (8.12)	8.52 (6.93)	-3.041	0.002	13.88 (8.20)	13.36 (5.54)	-0.385	0.700
Lymph node	13.71 (7.95)	8.54 (7.30)	-3.028	0.002	14.96 (8.68)	13.69 (4.60)	-0.456	0.648
Bone	8.18 (3.69)	9.50 (2.38)	-1.294	0.196	12.17 (6.80)	18.25 (3.21)	-1.782	0.075

IQR, interquartile range; PT, primary tumors; MT, metastatic tumors; All metastatic sites, including lymph node, bone, brain, pleural fluid and pleura.

In all metastatic sites, SUVmax was higher in PT than MT in PD-L1 low subgroup (11.39 (8.12) vs 8.52 (6.93), P = 0.002). No significant difference in SUVmax was observed between PT and MT in the PD-L1 high subgroup (13.88 (8.20) vs 13.36 (5.54), P = 0.700).

In lymph node, SUVmax was significantly elevated in PT compared to MT in PD-L1 low expression (13.71 (7.95) vs 8.54 (7.30), P = 0.002), whereas no correlation was observed in PD-L1 high cases (14.96 (8.68) vs 13.69 (4.60), P = 0.648).

In bone, there was no significant difference in SUVmax between PT and MT, in both PD-L1 low expression (8.18 (3.69) vs 9.50 (2.38), P = 0.196) and high cases (12.17 (6.80) vs 18.25 (3.21), P = 0.075).

## Discussion

In this study, we analyzed the heterogeneity of PD-L1 expression in PT and MT. In 598 patients and 456 ADC patients, a significantly higher prevalence of PD-L1 high expression was observed in MT relative to PT. However, no significant difference was observed in patients with SCC. This result might be attributed to the limited number of metastatic tumor (MT) cases in this subgroup, which included only 17 patients, with 7 showing PD-L1 high expression and 10 showing low expression. The association of male, larger tumor size (≥30 mm), and smoking history with PD-L1 high expression in our study were potentially linked to two key factors: the definition of the PD-L1 high expression cutoff and variations in baseline immune competence (17) among patients. In our multivariate logistic regression, SUVmax and bx site were related factors of high PD-L1 expression in both NSCLC and ADC. However, SUVmax was the sole related factor after Benjamini-Hochberg FDR correction.

Our findings were in agreement with some previous studies (10, 11, 18), but in contrast to others which reported higher PD-L1 expression in primary tumors (8, 9, 19, 20). The discrepancies between studies likely stem from the following factors: (1) Pathological heterogeneity: In NSCLC, the PD-L1 positive rates differ due to varying proportions of adenocarcinoma and squamous cell carcinoma, and these rates also vary among adenocarcinoma subtypes; (2) Heterogeneity in sample sources: for biopsies obtained via needle aspiration, the limited tissue sample may not fully represent the tumor and its microenvironment (21); (3) Variability in PD-L1 positivity thresholds: different studies have employed varying cut-off values (1%, 5%, or 50%). In our study, we also considered PD-L1 expression on tumor-infiltrating immune cells, referencing criteria used in prior clinical trials. Our PD-L1 positive scores on TC no less than 50% or IC no less than 10% was defined as high expression; (4) Composition of metastatic sites analyzed: Previous literature suggests that PD-L1 expression is higher in lymph nodes, pleural effusions, soft tissues, and adrenal metastases compared to primary tumors, whereas the opposite trend is observed for pleural, hepatic, cerebral, and bone metastases (10). In our cohort, however, PD-L1 high expression was significantly higher in lymph node, bone, and brain metastases, lower in malignant pleural effusions, and comparable in pleural metastases relative to the primary sites. Although the sample sizes for bone, brain, pleural effusion, and pleural metastases were limited—potentially introducing bias—the overall data still indicate that PD-L1 expression differs between primary tumors and metastatic tumors. Thus, varying proportions of metastatic lesion biopsies result in differences in PD-L1 expression.

Given that PET/CT provides a comprehensive, whole-body assessment of both primary and metastatic disease, we were prompted to compare the metabolic activity between PT and MT across different PD-L1 expression levels. Significantly higher SUVmax was associated with PD-L1 high expression in all but one subgroup, with the MT of SCC being the notable exception. Our results were in line with previous studies, in both NSCLC (14, 22–24) and ADC (25). Within the SCC metastatic subgroup, the analysis yielded a negative correlation between SUVmax and PD-L1 expression. However, this finding should be interpreted with caution due to the small number of patients in this subset, which increases the risk of a Type II statistical error. In further research, we classified PD-L1 expression into different categories (two categories, positive, TC/IC≥1%; two categories, positive, TC/IC≥5%; three categories, TC/IC< 1%, TC1-49% or IC1-9% and TC≥50% or IC≥10%) and explored its correlation with SUVmax. We still reached the conclusion that SUVmax was positively correlated with PD-L1 expression in all of those categories (shown in **Supplementary Figures 1**-**3**). These research results indicated that there was a stable positive correlation between SUVmax and PD-L1 expression.

Initial comparisons of SUVmax between PT and MT across different PD-L1 expression levels (considering all patients) yielded no significant differences. Recognizing that PT and MT data often originated from different individuals, we refined our approach to focus on paired samples within the same patient. In patients where PD-L1 status was determined from MT, comparative analysis of paired PT-MT metabolic activity revealed a distinct pattern: within the PD-L1 low group, the SUVmax of PT demonstrated a significantly higher than its paired MT. In contrast, no significant correlation was observed in the PD-L1 high group. This significant difference was the same to lymph node metastases. Previous study pointed out PD-L1 expression was linked to HIF-α and GLU1 (26). Collectively, two reasonable inferences were as following: First, in our cohort, PD-L1 low result in a metastasis (particularly lymph node) did not preclude the possibility of PD-L1 high expression in the corresponding primary tumor; Second, the relationship between PD-L1 expression and intra-patient metabolic heterogeneity (PT vs MT) appeared to be metastasis site-dependent, indicating distinct biological behaviors across different metastatic sites.

Our study elucidated the metabolic basis for the observed variations in PD-L1 detection across different biopsy sites. Utilizing whole-body PET/CT imaging facilitates the selection of optimal biopsy sites by providing metabolic reference data pertinent to clinical practice. It was plausible that metastatic tumors exhibiting metabolic profiles analogous to those of the primary tumors and readily accessible for biopsy may enhance clinical reference value. There are some limitations in the study: First, we lacked that PT and various MT from the same patient were subjected to pathological examination and metabolism, which allows for a more direct comparative analysis; Second, only SUVmax was analyzed in the present study; other quantitative PET metabolic metrics were not evaluated, including metabolic tumor volume (MTV) and total lesion glycolysis (TLG) (27); Third, it was a retrospective and single-center design with potential selection bias; Fourth, in our multivariate logistic regression, while reaching statistical significance driven by our relatively large sample size, the OR value was numerically close to 1, and their direct individual-patient clinical utility remains limited. This preliminary inference warrants validation in larger, prospective cohorts.

## Conclusions

Both PD-L1 expression and metabolism exhibited heterogeneity between primary tumors and their metastases. Compared to the primary tumors, different types of metastatic tumors showed variations not only in PD-L1 expression but also in their metabolic activity. Utilizing whole-body PET/CT imaging facilitates the selection of optimal biopsy sites by providing metabolic reference data pertinent to clinical practice.

## Data Availability

The raw data supporting the conclusions of this article will be made available by the authors, without undue reservation.
