# Supplementary material for: Unveiling the heterogeneity: PD-L1 expression and metabolic activity in primary vs. metastatic NSCLC, a retrospective single-center analysis
Source: Front Oncol. 2026 Oct 13;16:1966803. doi: 10.3389/fonc.2026.1966803 (PMC13709988; doi:10.3389/fonc.2026.1966803)
Supplement: Supplementary file 1 [file SupplementaryFile1.docx]

**Figure S1** SUVmax and PD-L1 expression(Positive, TC/IC≥1%) in primary and metastatic tumors: (A) In NSCLC, SUVmax of PD-L1 positive expression was significantly higher than that of PD-L1 negative expression, in both PT and MT; (B) In ADC, SUVmax of PD-L1 positive expression was significantly higher than that of PD-L1 negative expression, in both PT and MT; (C) In SCC, SUVmax was significantly elevated in PD-L1 positive compared to PD‑L1 negative cases, in both PT and MT.

**Figure S2** SUVmax and PD-L1 expression(Positive, TC/IC≥5%) in primary and metastatic tumors: (A) In NSCLC, SUVmax of PD-L1 positive expression was significantly higher than that of PD-L1 negative expression, in both PT and MT; (B) In ADC, SUVmax of PD-L1 positive expression was significantly higher than that of PD-L1 negative expression, in both PT and MT; (C) In SCC, SUVmax was significantly elevated in PD-L1 positive compared to PD‑L1 negative cases in both PT and MT.

**Figure S3** SUVmax and PD-L1 expression(Negative, TC/IC< 1%; Low, TC1‑49% or IC1-9%; High, TC≥50% or IC≥10%) in primary and metastatic tumors: (A) In NSCLC, SUVmax of PD-L1 low and high expression was significantly higher than that of PD-L1 negative expression, in both PT and MT; (B) In ADC, SUVmax of PD-L1 low and high expression was significantly higher than that of PD-L1 negative expression, in both PT and MT; (C) In SCC, SUVmax was significantly elevated in PD-L1 low and high expression compared to PD‑L1 negative cases, in both PT and MT.
